# Application of ImageJ program to the enumeration of *Orientia tsutsugamushi* organisms cultured in vitro

**DOI:** 10.1016/j.trstmh.2012.05.004

**Published:** 2012-10

**Authors:** Sontana Siritantikorn, Suthatip Jintaworn, Sansanee Noisakran, Yupin Suputtamongkol, Daniel H. Paris, Stuart D. Blacksell

**Affiliations:** a Department of Microbiology, Faculty of Medicine Siriraj Hospital, Mahidol University, Bangkok 10700 Thailand; b Mahidol-Oxford Tropical Medicine Research Unit, Faculty of Tropical Medicine, Mahidol University, Bangkok, 10400 Thailand; c Medical Biotechnology Research Unit, National Center for Genetic Engineering and Biotechnology, National Science and Technology Development Agency, Bangkok 10700, Thailand; d Department of Medicine, Faculty of Medicine Siriraj Hospital, Mahidol University, Bangkok 10700 Thailand

**Keywords:** ImageJ, Scrub typhus, Enumeration, Fluorescence, *Orientia tsutsugamushi*

## Abstract

The ImageJ program was applied to the enumeration of *Orientia tsutsugamushi* organisms in cell culture using indirect immunofluorescence assay (IFA). The highest correlation (r = 0.984) was observed between manual counting methods and the ImageJ program (MaxEntropy threshold algorithm). This software-based methodology is cheaper, more standardised and better reproducible than a manual-based approach.

## 1 Introduction

*Orientia tsutsugamushi* is an obligate intracellular bacterium and causative agent of scrub typhus. Multiplication of *O. tsutsugamushi* occurs in the cytoplasm of infected cells with a doubling time of between 9 and 18 h.^1^ The manual enumeration of *O. tsutsugamushi* examined under a microscope becomes difficult when a large number of particles exist in a microscopic field. The small size of *O. tsutsugamushi* (0.5–2 μm) usually makes manual counting difficult as numbers of organisms increase.

The ImageJ program is a Java-based open source image enumeration software package freely downloadable from the US National Institute of Health website (http://imagej.nih.gov/ij/). ImageJ has been used to enumerate malaria parasites on Giemsa-stained thick blood films and *Chlamydia* spp. inclusion bodies in cell culture by immunofluorescence.^2^, ^3^ Here we have applied ImageJ to counting of *O. tsutsugamushi.*

## 2 Materials and methods

### 2.1 Orientia tsutsugamushi propagation and indirect immunofluorescence visualisation

In this study, L929 cells were grown as monolayers on glass cover slips in 24 well culture plates. When 80% confluent, the cells were infected with a predetermined dilution of *O. tsutsugamushi* (isolate UT76) inoculum and incubated at 35 °C with 5% CO_2_ using maintenance media (5% FBS + RPMI 1640, (Gibco, Carlsbad, CA, USA)) for 8 hours. Following incubation, the infected cells were fixed and permeabilized in acetone for 10 min at −20 °C and allowed to air dry. Indirect immunofluorescence (IFA) was performed to visualize the intracellular *O. tsutsugamushi* organisms*.* The coverslips were incubated with pooled human serum (diluted 1:320 in PBS) from *O. tsutsugamushi* confirmed-patients at 37 °C for 30 min, washed twice with PBS, then further incubated with FITC-conjugated goat antihuman IgG (Gibco) diluted 1:40 in PBS for 30 min at 37 °C. The monolayer was then washed twice with PBS and the cells were counterstained with 0.00125% (w/v) Evans blue. The infected cells were visualized by epifluorescence microscopy (Nikon Eclipse 80i, Nikon Corp., Chiyoda-ku, Tokyo, Japan).

### 2.2 Digital image capture

Images of *O. tsutsugamushi* infected in cell culture were captured by digital camera (Nikon Digital Sight DS-5M-L1, Japan) at a 400× magnification. The method for enumeration of *O. tsutsugamushi* using ImageJ required the image file to be converted from RGB color to 8-bit grayscale.

### 2.3 ImageJ manual counting

The manual counting of the *O. tsutsugamushi* particles was performed using the built-in cell-counter plugin of the ImageJ program. After opening the image to be counted, the cell-counter plugin was opened (commands used: *Plugins* *>* *Analyze* *>* *Cell Counter*), ‘internalize’ and ‘Type 1’ selected. The *Orientia* particles were manually counted by the operator by moving the crosshairs over the particle and confirming the identity of the particle by clicking the mouse button. The number of *Orientia* particles selected was then displayed within the plugin.

### 2.4 ImageJ automated counting

Automated counting of the *O. tsutsugamushi* particles uses threshold algorithms to discriminate the features of interest from background. The threshold level is dependent on the algorithm selected and in this study Minimum, MaxEntropy, RenyiEntropy and Yen threshold algorithms^4^, ^5^, ^6^ were used however another twelve algorithms were assessed and found to be unsuitable for this application. To set the counting threshold following opening the selected image, the following commands *Image* *>* *Adjust* *>* *Threshold* *>* *select algorithm to be applied* *>* *Apply* were used and the image converted to a binary image by selecting *Process* *>* *Binary* *>* *Make binary*. *O. tsutsugamushi* particles were counted using the commands *Analyze* *>* *Analyze Particles*, with the the upper and lower limits for the particle size set at 0–infinity, selected to ‘Show outlines’ and checked box to ‘Summarize’. Each counted particle was outlined and numbered in a new window.

### 2.5 Manual and automated counting comparison

Twenty-five IFA image fields were digitally photographed and the images processed as described above. *O. tsutsugamushi* were counted manually (one operator) and by ImageJ using Minimum, MaxEntropy, RenyiEntropy and Yen threshold algorithms.^4^, ^5^, ^6^ Pearson's correlation was calculated between the manual counting method and each of the ImageJ algorithms to determine the most appropriate algorithm.

## 3 Results and Discussion

**Figure 1:**
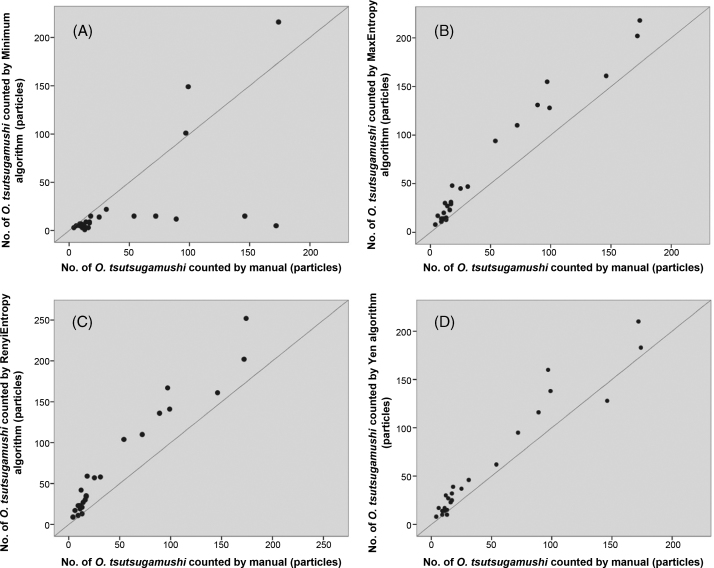
Correlations of manual versus by software-based (ImageJ program) enumeration of *Orientia tsutsugamushi* particles. The figure presents the correlations of results of manual versus software-based enumerations. The threshold algorithms used were: (A) Minimum, (B) MaxEntropy, (C) RenyiEntropy (D) Yen modalities. The MaxEntropy threshold algorithm achieved the highest correlation co-efficient (0.984).

Comparison between manual and ImageJ algorithms demonstrated strong, significantly (p < 0.05) positive correlations (Figure 1) for Yen (r = 0.969; p≤0.00005), MaxEntropy (r = 0.984; p≤0.00005), RenyiEntropy (r = 0.974; p≤0.00005) and to a lesser extent the Minimum algorithm (r = 0.612; p = 0.0012)).

Traditionally, the enumeration of viable *O. tsutsugamushi* organisms has employed several methodologies. The plaque assay for *O. tsutsugamushi* requires a minimum of 12–14 days of in vitro cultivation in cell culture until plaques can be observed.^1^, ^7^ A mouse model-based lethal dose (LD)_50_ method for quantifying *O. tsutsugamushi*^8^, ^9^ has been used for vaccine trials. Flow cytometry-based assays have been developed but are laborious and have limited accuracy.^10^, ^11^ The thymidine uptake assay uses uptake rates of radiolabeled thymidine incorporated into DNA during *O. tsutsugamushi* replication which is then converted to rates of *O. tsutsugamushi* production.^12^ This method is useful because it measures viable *O. tsutsugamushi* but is limited by the general measurement of the total ‘load’ of infection, rather than being discriminatory to the level of an individual bacterium. Recently, molecular techniques such as quantitative real-time PCR assays based on the *groEL*, 47 kDa and 16S rRNA genes of *O. tsutsugamushi* allow sensitive bacterial quantitation down to <5 copies/μl in an efficient, standardizable and cost-effective way.^13^, ^14^, ^15^ However, the manual count method based on direct visualisation of *O. tsutsugamushi* via Giemsa, Gimenez or immunofluorescence remains a widely used approach where detailed quantitative viable bacterial counts are accessible and/or required.^8^, ^10^, ^16^

This is the first study to describe a new and simple software-based method for quantification of *O. tsutsugamushi.* ImageJ comprises many image analysis capabilities, including functions for calculating area, measuring distances and counting. Cross-validation of software versus manual based counting methods resulted in high positive correlations for three discrimination algorithms of the ImageJ program, the best being the MaxEntropy threshold algorithm, however, RenyiEntropy and Yen algorithms would also be suitable given their high correlation values. Direct staining and visualization of organisms for counting can benefit greatly from the use of ImageJ software; also this method is less expensive and less laborious than other methods and is more rapid and reproducible than counting using manual microscopy methods. Therefore we suggest the application of the ImageJ program as an alternative method to manual quantification of *O. tsutsugamushi*. However, due to limitations of the study, the manual counting was only performed by a single operator and therefore we suggest that more extensive application of this method is required to explore applications and limitations.

## Authors’ contributions

SS, SDB, SN and YS designed the study protocol. SJ carried out the IFA and SS, SJ and SDB performed the analysis. SJ, SDB and DHP drafted the manuscript. All authors read and approved the final manuscript. SS and SDB are guarantors of the paper.

## Funding

None.

## Competing interests

None declared.

## Ethical approval

Not required.
